# A Comparison of the Electrophysiological and Anatomic Characteristics of Pacing Different Branches of the Left Bundle Conduction System

**DOI:** 10.3389/fcvm.2021.781845

**Published:** 2022-01-05

**Authors:** Xi Liu, Min Gu, Hong-Xia Niu, Xuhua Chen, Chi Cai, Junhan Zhao, Minsi Cai, Xiaohong Zhou, Michael R. Gold, Shu Zhang, Wei Hua

**Affiliations:** ^1^Cardiac Arrhythmia Center, Fuwai Hospital, National Center for Cardiovascular Diseases, Chinese Academy of Medical Sciences and Peking Union Medical College, Beijing, China; ^2^Cardiac Rhythm and Heart Failure Division, Medtronic plc, Minneapolis, MN, United States; ^3^Division of Cardiology, Medical University of South Carolina, Charleston, SC, United States

**Keywords:** left bundle branch pacing, left bundle trunk pacing, left bundle fascicular pacing, vectorcardiogram, visualization technique

## Abstract

**Introduction:** Left bundle branch pacing (LBBP) is a rapidly growing conduction system pacing technique. However, little is known regarding the electrophysiological characteristics of different types of LBBP. We aimed to evaluate the electrophysiological characteristics and anatomic lead location with pacing different branches of the left bundle branch.

**Methods:** Consecutive bradycardia patients with successful LBBP were enrolled and classified into groups according to the paced electrocardiogram and the lead location. Electrocardiogram, pacing properties, vectorcardiogram, and lead tip location were analyzed.

**Results:** Ninety-one patients were enrolled, including 48 with the left bundle trunk pacing (LBTP) and 43 with the left bundle fascicular pacing (LBFP). The paced QRS duration in the LBTP group was significantly shorter than that in the LBFP group (108.1 ± 9.9 vs. 112.9 ± 11.2 ms, *p* = 0.03), with a more rightward QRS transition zone (*p* = 0.01). The paced QRS area in the LBTP group was similar to that during intrinsic rhythm (35.1 ± 15.8 vs. 34.7 ± 16.6 μVs, *p* = 0.98), whereas in the LBFP group, the paced QRS area was significantly larger compared to intrinsic rhythm (43.4 ± 15.8 vs. 35.7 ± 18.0 μVs, *p* = 0.01). The lead tip site for LBTP was located in a small fan-shaped area with the tricuspid valve annulus summit as the origin, whereas fascicular pacing sites were more likely in a larger and more distal area.

**Conclusions:** Pacing the proximal left bundle main trunk produced better electrical synchrony compared with pacing the distal left bundle fascicles. A visualization technique can facilitate achieving LBTP.

## Introduction

Left bundle branch pacing (LBBP) is a conduction system pacing (CSP) technique, which overcomes some of the limitations with His bundle pacing (HBP) (1). Unlike the relatively small distribution of the His bundle (HB) region, the left bundle branch (LBB) is a major extension of the HB with a larger anatomical distribution. It is composed of a short and thick left bundle main trunk and two main fascicles, the left anterior fascicle (LAF) and left posterior fascicle (LPF) (2). Theoretically, pacing any parts of the LBB can capture the left sided conduction system. However, the impact of pacing site of the LBB on electrophysiological characteristics and ventricular synchrony is not well-studied.

Recently, the QRS area obtained by the 3-dimensional (3D) vectorcardiography (VCG) has emerged as a reliable index to evaluate ventricular synchrony (3). Compared with the traditional electrocardiogram (ECG), the VCG contains 3D information of the electrical forces, which provides additional valuable information to the ECG. Previous studies showed that the QRS area predicted cardiac resynchronization therapy (CRT) response better than the QRS duration, and was strongly associated with clinical outcomes (4, 5).

The aim of the present study was to compare electrophysiological characteristics and ventricular synchrony of different pacing sites of the LBB. In addition, a novel visualization technique was used to correlate anatomic location with paced LBB morphology to help guide pacing different components of the LBB (6).

## Materials and Methods

**Figure 1:**
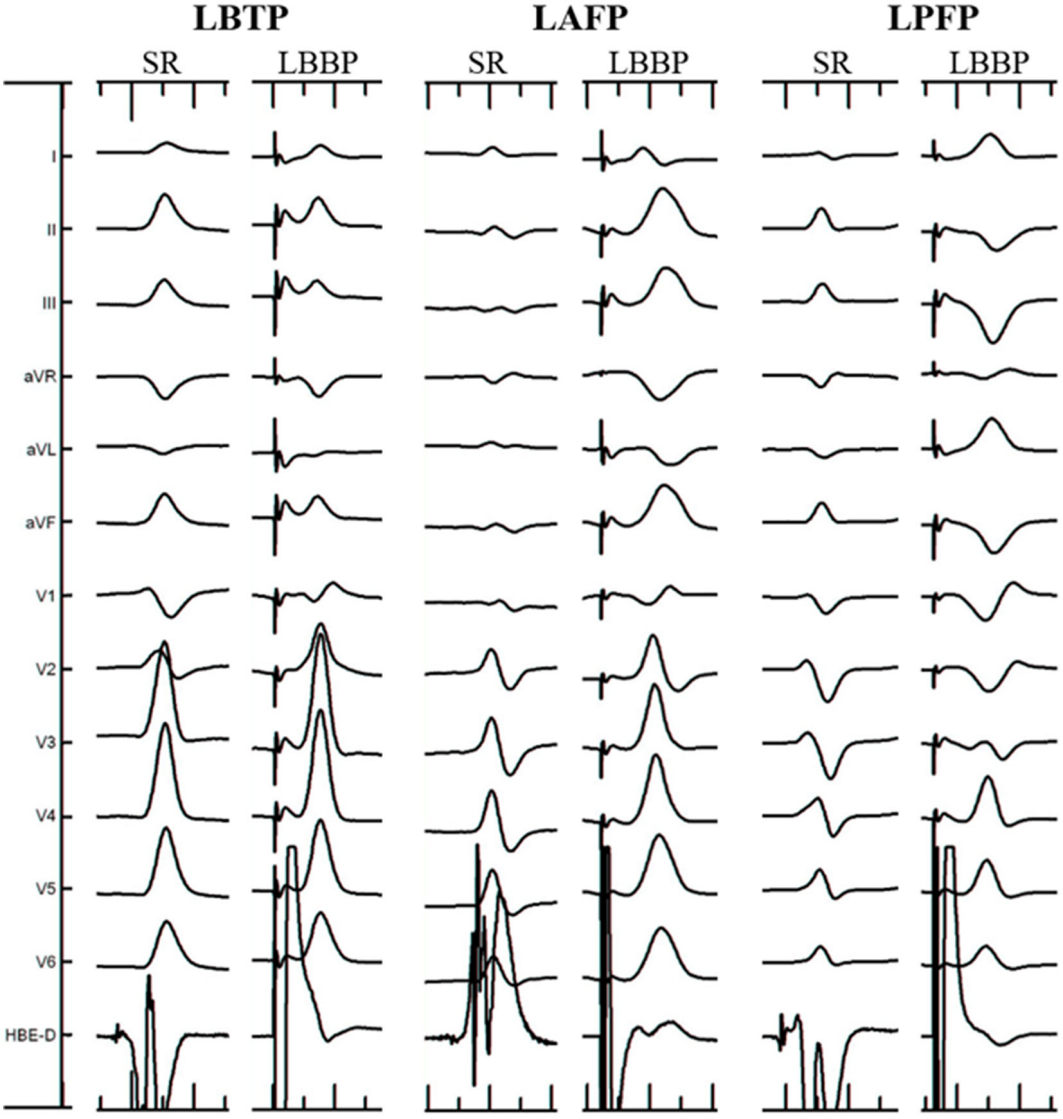
Electrocardiographic characteristics of different ECG types. LAFP, left anterior fascicular pacing; LBBP, left bundle branch pacing; LBTP, left bundle trunk pacing; LPFP, left posterior fascicular pacing; SR, sinus rhythm.

**Figure 2:**
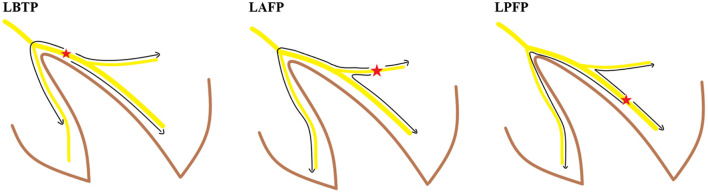
Activation sequence of the conduction system in different ECG types. LAFP, left anterior fascicular pacing; LBTP, left bundle trunk pacing; LPFP, left posterior fascicular pacing.

Consecutive patients who underwent LBBP with bradycardia indications including sinus node dysfunction (SND) or atrioventricular block (AVB) from Fuwai Hospital (Beijing, China) were analyzed. Patients were classified into two groups including the left bundle trunk pacing (LBTP) and the left bundle fascicular pacing (LBFP) group. The latter group included those with either a paced LAF or LPF morphology (Figures 1, 2). Patients were excluded if they had a native QRS duration longer than 120 ms, including left bundle branch block (LBBB), right bundle branch block (RBBB), and non-specific intraventricular conduction disturbance (NIVCD). This study was approved by the Ethics Committee of Fuwai Hospital and all patients submitted the written informed consent.

### Implantation Procedure

LBBP implantation was performed using the Select Secure 3830 pacing lead (Medtronic Inc., Minneapolis, MN) and the fixed-curve C315 HIS sheath (Medtronic Inc., Minneapolis, MN). The implantation procedure was performed as previously described (6). Successful LBBP was assumed in patients whose paced ECG morphology in lead V1 showing a RBBB pattern and also met at least one of the following three criteria: (1) recording of an LBB potential; (2) left ventricular activation time (LVAT) remained short and constant (<80 ms) at different pacing outputs or was abruptly shortened (≥10 ms) at high output; (3) demonstration of selective LBB capture.

### LBB Lead Tip Location Evaluation

**Figure 3:**
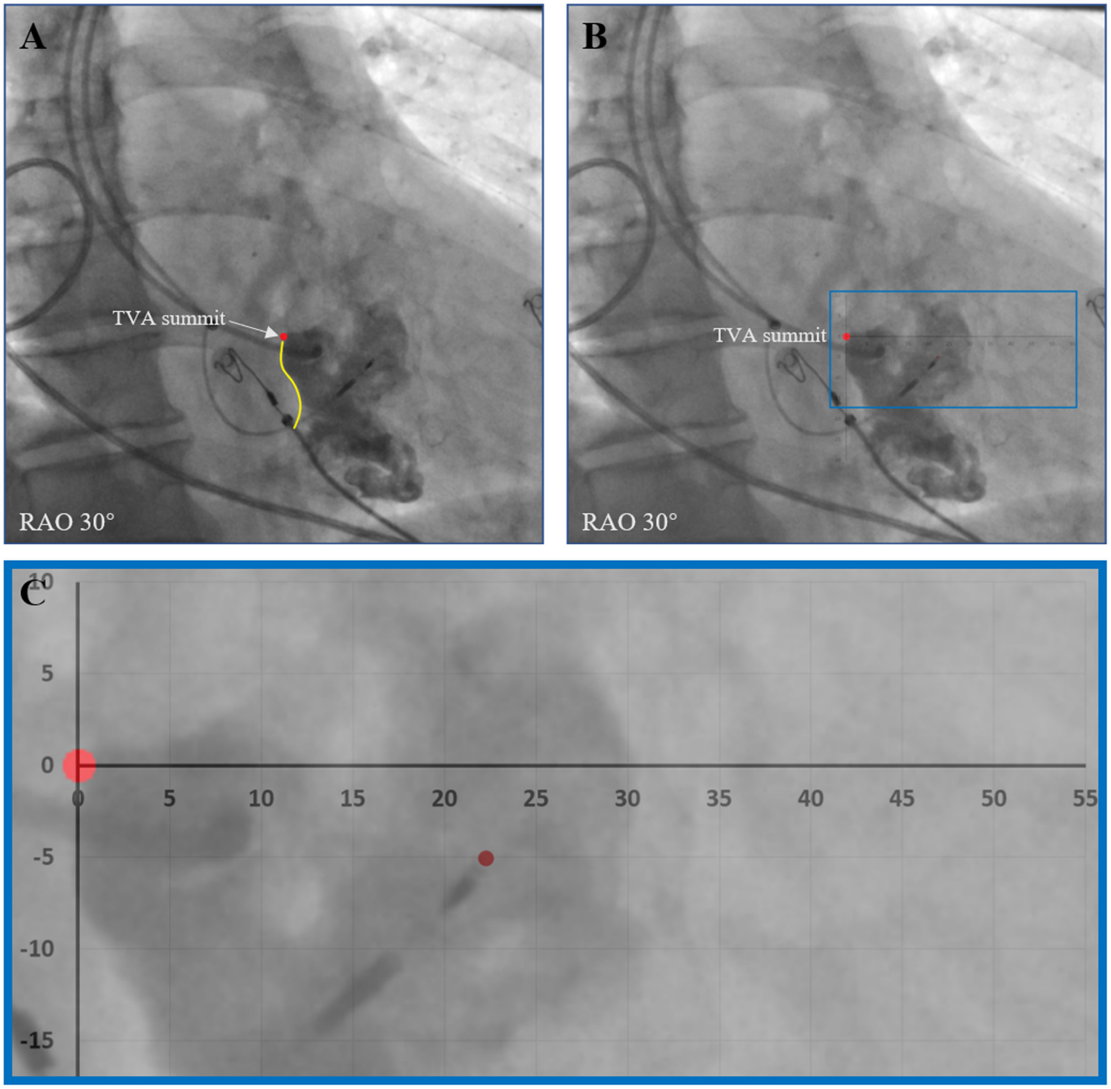
Evaluation of the positional relationship between the LBB lead tip and the TVA. **(A)** After the LBB lead was deployed, visualization of the TVA was performed to show the TVA summit. **(B)** The horizontal and vertical distances between the LBB lead tip and the TVA summit were measured offline. **(C)** An enlarged view of the measurement. LBB, left bundle branch; RAO, right anterior oblique; TVA, tricuspid value annulus.

During the procedure, right ventriculography was performed with an injection below the root of the tricuspid septal leaflet with 10–15 ml contrast medium through C315 HIS sheath imaged in the right anterior oblique 30° (RAO 30°) fluoroscopic view. Then the fluoroscopic image of the tricuspid value annulus (TVA) was saved and served as a marker to help locate the target LBB region according to the positional relationship between the TVA and the LBB as revealed by our previous study. The target LBB area included area 1 and area 2. Area 1 was defined as a fan-shaped area drawn from the TVA summit with a radius of 15–35 mm and angle ranging from + 10 to −30°, area 2 was defined as a more distal fan-shaped area with a radius of 35–50 mm and angle ranging from + 10 to −60°. After the LBB lead was deployed, visualization of the TVA was performed again through another C315 HIS sheath to finally confirm the lead tip location (6). The horizontal and vertical distances between the LBB lead tip and the TVA summit were measured offline in each of the patients (Figure 3).

### ECG Criteria for Determining the LBB Lead Tip Location

The criteria for determining the electrophysiological classification of LBB lead tip location was based on previous studies of the ECG morphology of left bundle fascicular block and fascicular ventricular arrhythmia (Figure 1) (7–10). The types of ECG criteria include: (1) LBTP: RBBB pattern; paced QRS morphology similar to sinus rhythm; (2) left anterior fascicular pacing (LAFP): RBBB pattern; dominant S wave in leads I and aVL; dominant R wave in leads II, III, and aVF; right-axis deviation; (3) left posterior fascicular pacing (LPFP): RBBB pattern; dominant R wave in leads I and aVL; dominant S wave in leads II, III, and aVF; left-axis deviation. Patients who met the ECG criteria of LBTP were classified into the LBTP group, whereas patients who meet the ECG criteria of LAFP or LPFP were classified into the LBFP group. All ECGs were evaluated by two independent experienced electrophysiologists blinded to the anatomic location. In cases of discrepancy between reviewers, a third electrophysiologist provided adjudication.

### ECG and VCG Analysis

For ECG analysis, the 12-lead ECG were recorded using an electrophysiology workstation (Bard, Boston Scientific, Lowell, MA). The QRS duration and QRS transition zone were recorded before and after the procedure. The LVAT was defined as the interval from the pacing stimulus to the R-wave peak of the QRS complex in leads V5-V6.

**Figure 4:**
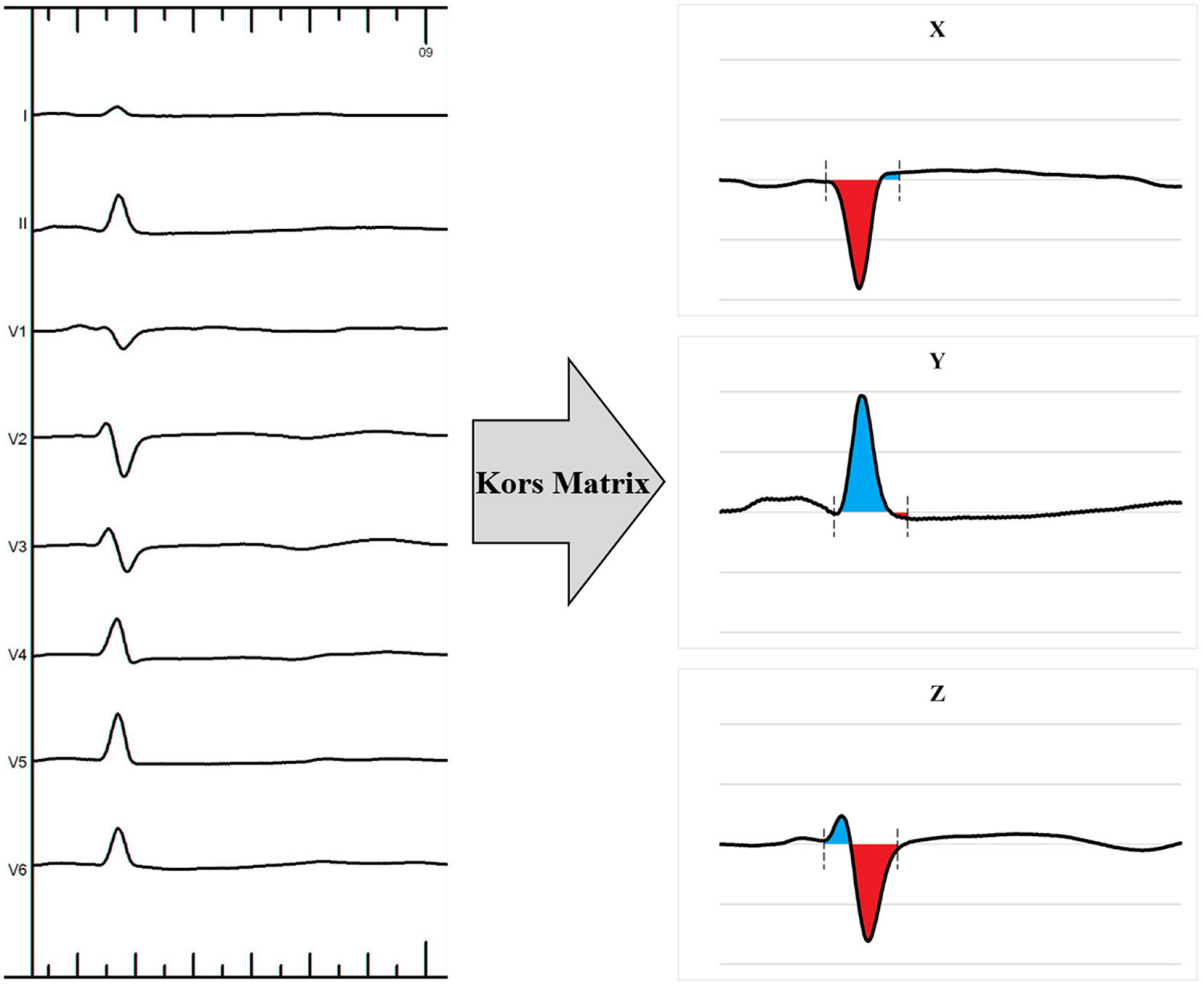
Example of a 12-lead electrocardiogram transformed into a 3-dimensional vectorcardiogram using the Kors matrix.

For VCG analysis, the customized MATLAB software (MathWorks Inc., Natick, MA) was used to convert the 12-lead ECG into the 3 orthogonal VCG leads (X, Y, and Z) using the Kors conversion matrix as described previously (Figure 4) (3, 11, 12). This matrix was based on a learning set from the Common Standards for Electrocardiography multilead library, including both patients and healthy individuals, and was generated by multiple linear regression. The VCG was synthesized by analyzing eight independent ECG leads (two limb leads and all six precordial leads) retrieved from the 12-lead ECG by the Kors conversion matrix. QRS area, which represents the extent of the unopposed electrical forces during ventricular activation, was calculated as the combined area under the QRS complex in the calculated vectorcardiographic X, Y, and Z leads [QRS area = (QRS area, x^2^ + QRS area, y^2^ + QRS area, z^2^)^1/2^].

### Data Collection and Follow-Up

Baseline data including the demographic characteristics, indications for pacemaker implantation and echocardiographic measurements were collected at enrollment. Pacing parameters including capture threshold, R-wave amplitude, and impedance were recorded during the procedure and at 12-month follow-up. Procedural related complications including loss of capture, lead septal perforation, and lead dislodgement were tracked during follow-up.

### Statistical Analysis

Continuous variables are presented as mean ± standard deviation or as median (interquartile range), and categorical variables are expressed as frequencies or percentages. Independent two sample *t*-test or analysis of variance (ANOVA) test are used to compare the differences between groups if the data are normally distributed. Wilcoxon signed rank test or Kruskal-Wallis test are performed for data that are not normally distributed. Chi square or Fisher's exact test are used to compare categorical variables. For within patients' comparisons of continuous variables, paired *t*-test are used for normally distributed data and Wilcoxon signed rank test for non-normally distributed data. A two-sided *P* < 0.05 is considered statistically significant. All statistical analyses are performed using the SPSS Statistics version 22.0 (IBM Corporation, Armonk, NY).

## Results

### Baseline Characteristics Among Groups

From April 2018 to January 2020, 127 patients successfully underwent LBBP implantation for bradycardia indications. Among this group, 36 patients were excluded for having underlying QRS prolongation, including 17 for LBBB, 11 for RBBB, and 8 for NIVCD. Accordingly, a total of 91 patients were included in the analysis. There were 48 patients classified into the LBTP group and 43 to the LBFP group. The LBFP group included 14 patients with LAFP and 29 with LPFP. There were no significant differences in baseline demographics, pacing indications, ECG, VCG, and echocardiographic measurements between patients with LAFP and LPFP (Table 1). These subgroups were pooled because these were relatively small cohorts that paced fascicles of the LBB. Similarly, there were no differences in baseline characteristics between the LBTP and LBFP groups (Table 1).

**Table 1:** Baseline characteristics between groups.

	**LBTP group (*n* = 48)**	**LBFP group (*****n*** **= 43)**			***P*** **-values**	
		**Total (*n* = 43)**	**LAFP (*n* = 14)**	**LPFP (*n* = 29)**	**LAFP vs. LPFP**	**LBTP vs. LBFP**
**Demographics**						
Age (years)	58.2 ± 19.6	58.1 ± 16.6	59.1 ± 12.0	57.6 ± 18.6	0.78	0.75
Male	24 (50.0%)	22 (51.2%)	7 (50.0%)	15 (51.7%)	0.92	0.91
**Comorbidities**						
Hypertension	27 (56.3%)	22 (51.2%)	6 (42.9%)	16 (55.2%)	0.45	0.63
Diabetes mellitus	6 (12.5%)	6 (14.0%)	3 (21.4%)	3 (10.3%)	0.37	0.84
Coronary artery disease	10 (20.8%)	8 (18.6%)	2 (14.3%)	6 (20.7%)	0.93	0.79
**Indications**					0.45	0.67
SND	18 (37.5%)	18 (41.9%)	7 (50.0%)	11 (37.9%)		
AVB	30 (62.5.0%)	25 (58.1%)	7 (50.0%)	18 (62.1%)		
**Baseline ECG**						
QRS duration (ms)	94.8 ± 9.6	94.3 ± 9.3	92.9 ± 9.0	95.0 ± 9.5	0.49	0.83
QRS transition zone	4.0 (3.5, 4.5)	4.0 (3.5, 4.5)	4.0 (3.5, 5.0)	4.0 (3.5, 4.0)	0.07	0.90
**Baseline VCG**						
QRS area (μVs)	34.7 ± 16.6	35.7 ± 18.0	35.4 ± 20.6	35.8 ± 17.0	0.67	0.95
**Echocardiography**						
LVEF (%)	61.1 ± 6.2	60.4 ± 6.2	59.3 ± 6.7	61.0 ± 6.0	0.46	0.69
LVEDD (mm)	49.8 ± 6.3	48.7 ± 4.8	48.9 ± 4.2	48.6 ± 5.1	0.85	0.56

*AVB, atrioventricular block; ECG, electrocardiogram; LAFP, left anterior fascicular pacing; LBFP, left bundle fascicular pacing; LBTP, left bundle trunk pacing; LPFP, left posterior fascicular pacing; LVEDD, left ventricular end-diastolic diameter; LVEF, left ventricular ejection fraction; SND, sinus node dysfunction; VCG, vectorcardiogram*.

### Electrophysiological Characteristics Among Groups

As shown in Table 2, the paced QRS duration in the LBTP group was significantly narrower than that in the LBFP group (108.1 ± 9.9 vs. 112.9 ± 11.2 ms, *p* = 0.03). In addition, the QRS transition zone in the LBTP group was more rightward than that in the LBFP group (*p* = 0.01). No significant differences were observed in LVAT between two groups (68.9 ± 6.4 vs. 67.7 ± 5.6 ms, *p* = 0.35).

**Table 2:** Procedural outcomes between groups.

	**LBTP group**	**LBFP group**	***P*-value**
	**(*n* = 48)**	**(*n* = 43)**	
**Lead tip distribution**			
Within area 1	36 (75.0%)	7 (16.3%)	<0.01
Within area 2	10 (20.8%)	35 (81.4%)	<0.01
**ECG parameters**			
Paced QRS duration (ms)	108.1 ± 9.9	112.9 ± 11.2	0.03
LVAT (ms)	68.9 ± 6.4	67.7 ± 5.6	0.35
QRS transition zone	1.3 (1.0, 1.5)	1.5 (1.5, 2.0)	0.01
**VCG parameters**			
QRS area (μVs)	35.1 ± 15.8	43.4 ± 15.8	0.01
**Pacing parameters**			
Capture threshold (V/0.4 ms)	0.8 ± 0.2	0.8 ± 0.3	0.97
R-wave amplitude (mV)	11.1 ± 4.4	10.0 ± 3.9	0.21
Impedance (Ω)	674.1 ± 130.9	677.5 ± 133.6	0.83
**Parameters at follow-up**			
Capture threshold (V/0.4 ms)	0.7 ± 0.2	0.7 ± 0.2	0.83
R-wave amplitude (mV)	11.8 ± 4.2	11.0 ± 3.9	0.49
Impedance (Ω)	478.9 ± 80.0	489.3 ± 73.4	0.39
**Echocardiography at follow-up**			
LVEF (%)	61.2 ± 5.1	60.8 ± 5.08	0.65
LVEDD (mm)	49.6 ± 5.6	48.7 ± 6.0	0.38

*ECG, electrocardiogram; LBFP, left bundle fascicular pacing; LBTP, left bundle trunk pacing; LVAT, left ventricular activation time; LVEDD, left ventricular end-diastolic diameter; LVEF, left ventricular ejection fraction; VCG, vectorcardiogram*.

**Figure 5:**
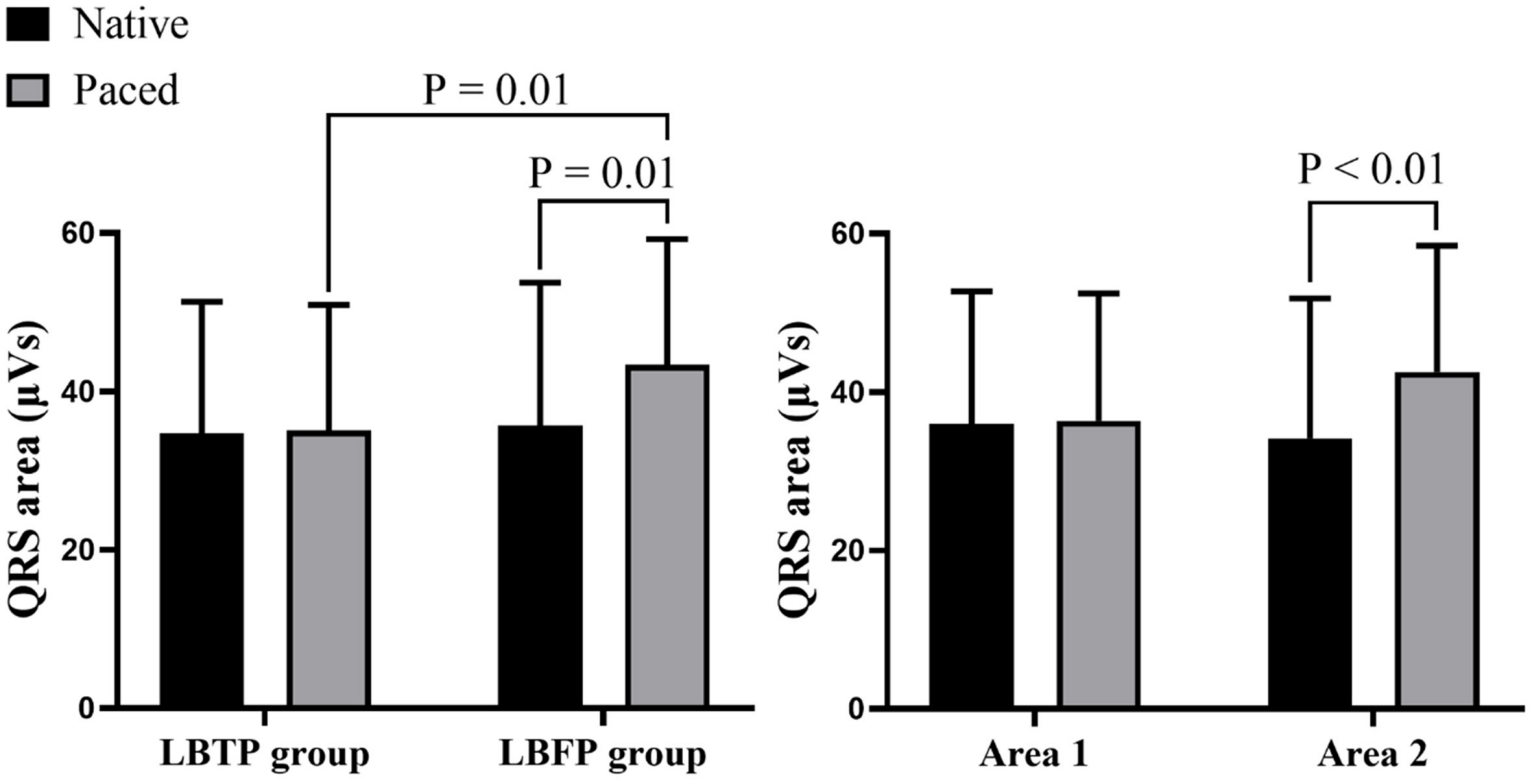
Comparison of the QRS area between different groups and different areas. LBFP, left bundle fascicular pacing; LBTP, left bundle trunk pacing.

VCG analysis showed that the paced QRS area in the LBTP group was similar to that during intrinsic rhythm (35.1 ± 15.8 vs. 34.7 ± 16.6 μVs, *p* = 0.98), whereas in the LBFP group, the paced QRS area was significantly larger compared to intrinsic ventricular activation (43.4 ± 15.8 vs. 35.7 ± 18.0 μVs, *p* = 0.01) (Figure 5). Paced QRS area was larger for the LBFP group compared with the LBTP group (43.4 ± 15.8 vs. 35.1 ± 15.8 μVs, *p* = 0.01) (Table 2; Figure 5).

### Lead Tip Distribution Among Groups

**Figure 6:**
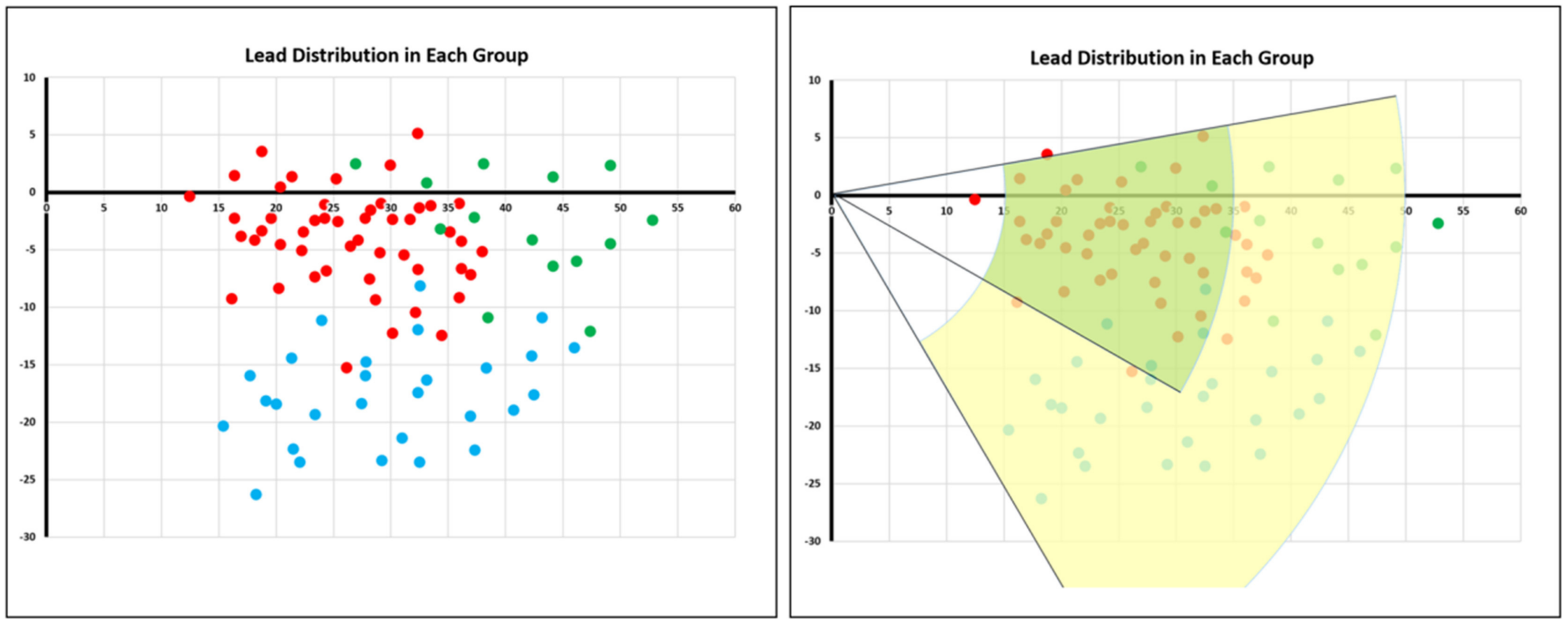
Lead tip distribution in different electrocardiogram types. The red dots represented patients with LBTP, the green dots represented patients with LAFP, and the blue dots represented patients with LPFP. The green area termed area 1 was a fan-shaped area drawn from the TVA summit with the radius from 15 to 35 mm and the angle range from + 10 to −30 degrees. The yellow area termed area 2 was a fan-shaped area with the radius from 35 to 50 mm and the angle range from + 10 to −60 degrees. LAFP, left anterior fascicular pacing; LBTP, left bundle trunk pacing; LPFP, left posterior fascicular pacing; TVA, tricuspid value annulus.

As shown in Table 2, the proportion of patients in the LBTP group with the lead tip in Area 1 was significantly higher than that in the LBFP group (75.0 vs. 16.3%, *p* < 0.01). Conversely, while the proportion of patients with the lead tip within area 2 was significantly lower (20.8 vs. 81.4%, *p* < 0.01). The positional relationship between the LBB lead tip and the TVA summit in each ECG type is shown in Figure 6, and the lead tip distribution of different ECG type in different areas is presented in Table 3. Overall, 97% (88 of 91) of patients had the lead tip within a fan-shaped area drawn from the TVA summit with the radius from 15 to 50 mm and the angle range from + 10 to −60 degrees (area 1 or area 2). Nearly half of the patients (43 of 91, 47%) had the lead tip within area 1. Among these patients, 84% (36 of 43) were classified into the LBTP group. The majority of patients (35 of 45, 78%) with lead tips in area 2 were classified into the LBFP group. Among the subgroup of LBFP, both LAFP and LPFP most commonly had the lead tip within area 2 (71 and 86%, respectively, *p* = 0.45). However, the overlapping distribution of the lead tip in different ECG types in area 2 makes it difficult to distinguish them by specific positioning method. In general, LAFP was more likely located in the upper part of the area 2, whereas LPFP was located in the lower part of the area 2 (Figure 6).

**Table 3:** Lead tip distribution in each ECG type.

	**LBTP**	**LAFP**	**LPFP**	**Total**
Area 1	36	3	4	43
Area 2	10	10	25	45
Other area	2	1	0	3
Total	48	14	29	91

*LAFP, left anterior fascicular pacing; LBTP, left bundle trunk pacing; LPFP, left posterior fascicular pacing*.

Further analysis between area 1 and area 2 showed that the patients having the lead tip within area 1 had a similar paced QRS area compared with their intrinsic rhythm (36.4 ± 16.1 vs. 35.9 ± 16.8 μVs, *p* = 0.75), whereas patients with the lead tip in area 2 had a significantly increased QRS area (42.6 ± 15.9 vs. 34.2 ± 17.7 μVs, *p* < 0.01) (Figure 5).

### Twelve-Month Follow-Up

No significant differences were observed between implantation and 12-month follow-up for electrical parameters of the lead as shown in Table 2. Similarly, echocardiographic measurements including LVEDD and LVEF were similar at follow-up (Table 2). One patient in both the LBTP and LBFP groups had a lead septal perforation during the procedure. The lead was immediately repositioned with no post-implant adverse effects. One patient in the LBFP group had lead dislodgement during follow-up, so a right ventricular pacing (RVP) lead was placed.

## Discussion

LBBP is a rapidly increasing conduction system pacing modality. In the present study, we evaluated the electrophysiological characteristics of pacing different parts of the LBB by comparing the paced ECG and VCG parameters. The primary findings of our study were that pacing the left bundle main trunk achieved narrower paced QRS duration than pacing the left bundle fascicles, and that the QRS transition zone was more rightward. Moreover, LBTP had a QRS area similar to intrinsic rhythm, whereas in the LBFP group, the QRS area was significantly larger than during intrinsic rhythm. These observations indicate worse ventricular synchrony. Furthermore, imaging showed that most patients with the lead tip within area 1 had LBTP. Fascicular pacing was noted more commonly in a broad and more distal area (area 2), which was associated with an increased paced QRS area. Since the paced QRS morphology cannot be assessed until the lead is deployed deep in the septum, the imaging technique helps to minimize repeat lead repositioning which are associated with high risk of perforation or other complications, as well as to achieve LBTP.

### Physiological Pacing in Bradycardia Patients

HBP is the most physiological pacing modality, as it activates the most proximal part of the native conduction system to achieve normal ventricular activation sequence (13). However, locating the HB can be challenging due to the small region of the HB. Moreover, the pacing parameters of HBP are less stable compared to traditional RVP with frequent high pacing thresholds (14). LBBP can overcome some of the limitations exists in HBP, and is considered as an alternative CSP technique (1). Anatomically, the LBB is divided into several parts. The main trunk of the LBB is usually short and thick, after a short path, it gives rise to its two main fascicles including a thin LAF and a wider LPF (2). However, little is known regarding the pacing characteristics at different LBB sites.

### Evaluation of Ventricular Synchrony

The paced ECG QRS duration is a commonly used index to evaluate ventricular depolarization. In general, a longer paced QRS duration is considered to represent worse ventricular synchrony. However, the paced QRS complex is a reflection of total ventricular activation. In patients who achieve CSP, pacing at any part of the conduction system can achieve relatively rapid ventricular activation, thus generate a similar paced QRS duration. This makes it difficult to compare the subtle differences between pacing sites. In a previous study of pacing different branches of the left bundle conduction system in a different cohort of patients, it was shown that the paced QRS duration were similar for different locations (9). In the present study with a larger sample size and different grouping method, the results shows that the paced QRS duration in the LBFP group was longer than that in the LBTP group, suggesting worse ventricular synchrony. Both studies showed that LPFP was more common than LAFP, likely reflecting the size of these fascicles. It should be noticed that, though pacing different parts of the LBB produced different ventricular synchrony, the overall conduction velocity is relatively fast due to the capture of the conduction system, so the absolute differences in paced QRS duration were relatively small.

The paced QRS area calculated by the VCG has emerged as a more sensitive measure of dyssynchronous electrical activation. The VCG contains more complete information on electrical forces, as the QRS area calculated by the VCG is the combined area under the QRS complex and represents the extent of unopposed electrical forces during ventricular activation (15). Previous studies showed the better predictive value of the QRS area than traditional QRS duration for echocardiographic response and clinical outcomes in CRT eligible patients (4, 5). In the present study, the paced QRS area in the LBFP group was significantly larger than during intrinsic rhythm or compared with LBTP, which further supports that ventricular synchrony is impaired with LBFP.

### Ideal LBBP Location

Theoretically, pacing the proximal main trunk of the LBB should result in better cardiac synchrony compared with pacing the left bundle fascicles. LBTP preserves left ventricular synchrony by sequentially activating each segment of the left ventricle. Moreover, among patients without heart block, retrograde activation of the RBB can rapidly activate the right ventricle with less time delay, thus maintaining interventricular synchrony and potentially achieve more normal right ventricular synchrony (Figure 2). In contrast, pacing distal LBB fascicles leads to different ventricular activation sequences, thus resulting in impaired left ventricular synchrony, reflected in part by changes in paced QRS axis (2). Moreover, the longer distance of retrograde activation of RBB by LBBP may exacerbate delayed right ventricular activation, leading to significantly decreased interventricular synchrony (Figure 2) (16).

The present study shows that ventricular synchrony evaluated by both QRS duration and VCG is optimized and more physiologic by pacing the main trunk of the LBB conduction system. In addition, the superiority of pacing the proximal LBB was verified by the significantly different QRS area between pacing in proximal area 1 and distal area 2 defined by our visualization technique. All of these findings support pacing the proximal left bundle main trunk instead the distal LBB fascicles when possible. The visualization technique used in this study can facilitate activating this area, which occurred more commonly in the present study (53%) compared previously using the traditional fluoroscopic approach (25%) (9).

### Clinical Perspectives

The findings of this study show the superiority of pacing the proximal left conduction system, as defined by either the ECG (left bundle main trunk) or the fluoroscopic lead tip location (area 1). While the present study was performed in a bradycardia population with a normal left ventricular function, providing the optimal LBBP would be crucial in heart failure patients who have left ventricular dyssynchrony and may benefit even more from LBTP. The visualization technique used in this study for LBBP lead deployment shortens procedural and fluoroscopic durations (6). Moreover, it facilitates achieving LBTP and reduces the need for repositioning to achieve LBBP. Finally, while LBTP achieves more physiologic activation compared with LBFP, CSP is superior to right ventricular pacing, with regard to paced QRS duration and ventricular synchrony, regardless of whether it is HBP, LBTP or LBFP.

## Limitations

The present study should be interpreted in light of certain methodological limitations. First, this was a single center study with a relatively small sample size. Second, only patients with a normal conduction system were evaluated. In other patient populations, the ventricular activation pattern may be different, and hence the results in this study may not be generalized to patients with conduction block. Third, the only acute measures of activation and electrical synchrony were assessed, and the absolute differences were relatively small. Whether these small differences are clinically significant will have to be shown in randomized clinical trials.

## Conclusions

When performing LBBP, pacing the proximal left bundle main trunk produced optimal ventricular synchrony than pacing the distal LBB fascicles. With the guidance of a visualization technique, LBTP is facilitated to help maintained ventricular synchrony.

## Data Availability Statement

The raw data supporting the conclusions of this article will be made available by the authors, without undue reservation.

## Ethics Statement

The studies involving human participants were reviewed and approved by the Ethics Committee of Fuwai Hospital. The patients/participants provided their written informed consent to participate in this study.

## Author Contributions

WH, SZ, MGo, XZ, and XL contributed to the study conception and design. XL, MGu, H-XN, and XC performed pacemaker implantation. XL, CC, JZ, and MC performed data collection and analysis. XL and MGu wrote the first draft of the manuscript. All authors commented on previous versions of the manuscript and read and approved the final manuscript.

## Funding

This work was supported by National Natural Science Foundation of China (Grant number: 82070349), Peking Union Medical College Youth Fund and Fundamental Research Funds for the Central Universities (Grant number: 3332019047, and 2017320006) and Innovation Funds for Graduate Students of Peking Union Medical College (Grant number: 2019-1002-33).

## Conflict of Interest

XZ is an employee of Medtronic. MGo receives consulting fees from Medtronic and Boston Scientific. The remaining authors declare that the research was conducted in the absence of any commercial or financial relationships that could be construed as a potential conflict of interest.

## Publisher's Note

All claims expressed in this article are solely those of the authors and do not necessarily represent those of their affiliated organizations, or those of the publisher, the editors and the reviewers. Any product that may be evaluated in this article, or claim that may be made by its manufacturer, is not guaranteed or endorsed by the publisher.
